# Bilateral lung volume reduction surgery outperforms the unilateral approach in functional improvement

**DOI:** 10.1093/icvts/ivae169

**Published:** 2024-10-01

**Authors:** Özlem Okumus, Gernot Seebacher, Daniel Valdivia, Alexis Slama, Kaid Darwiche, Rüdiger Karpf-Wissel, Johannes Wienker, Stephane Collaud, Sandra Kampe, Balazs Hegedüs, Clemens Aigner

**Affiliations:** Department of Thoracic Surgery, University Medicine Essen—Ruhrlandklinik, University of Duisburg-Essen, Essen, Germany; Department of Thoracic Surgery, Cologne-Merheim Hospital, Witten/Herdecke University, Cologne, Germany; Department of Thoracic Surgery, University Medicine Essen—Ruhrlandklinik, University of Duisburg-Essen, Essen, Germany; Department of Thoracic Surgery, Vienna Healthcare Group, Clinic Floridsdorf, Vienna, Austria; Department of Thoracic Surgery, University Medicine Essen—Ruhrlandklinik, University of Duisburg-Essen, Essen, Germany; Department of Thoracic Surgery, Klinikum Bielefeld, Bielefeld, Germany; Department of Thoracic Surgery, University Medicine Essen—Ruhrlandklinik, University of Duisburg-Essen, Essen, Germany; Department of Thoracic Surgery, Medical University of Vienna, Vienna, Austria; Section of Interventional Pneumology, Department of Pneumology, University Medicine Essen—Ruhrlandklinik, University of Duisburg-Essen, Essen, Germany; Section of Interventional Pneumology, Department of Pneumology, University Medicine Essen—Ruhrlandklinik, University of Duisburg-Essen, Essen, Germany; Section of Interventional Pneumology, Department of Pneumology, University Medicine Essen—Ruhrlandklinik, University of Duisburg-Essen, Essen, Germany; Department of Thoracic Surgery, University Medicine Essen—Ruhrlandklinik, University of Duisburg-Essen, Essen, Germany; Department of Thoracic Surgery, Cologne-Merheim Hospital, Witten/Herdecke University, Cologne, Germany; Department of Anesthesiology, University Medicine Essen-Ruhrlandklinik, University of Duisburg-Essen, Essen, German; Department of Thoracic Surgery, University Medicine Essen—Ruhrlandklinik, University of Duisburg-Essen, Essen, Germany; Department of Thoracic Surgery, University Medicine Essen—Ruhrlandklinik, University of Duisburg-Essen, Essen, Germany; Department of Thoracic Surgery, Medical University of Vienna, Vienna, Austria

**Keywords:** Chronic obstructive pulmonary disease, Lung volume reduction surgery, Pulmonary emphysema, Unilateral, Bilateral

## Abstract

**OBJECTIVES:**

Lung volume reduction surgery (LVRS) is an established treatment approach for patients with severe pulmonary emphysema, enhancing lung function and quality of life in selected patients. Functional benefits and outcomes after uni- versus bilateral lung volume reduction remain a topic of debate.

**METHODS:**

A retrospective analysis of patients undergoing LVRS from January 2018 to October 2022 was conducted. After encouraging initial results, the standard unilateral LVRS approach was switched to bilateral. The goal of this study was to assess the impact on functional outcomes at 3 and 6 months post-surgery compared to preoperative levels for the uni- versus the bilateral approach.

**RESULTS:**

A total of 83 patients were included (43 bilateral, 40 unilateral). Baseline demographic and functional parameters were comparable between groups. The most common complication was prolonged air leak in 19 patients (11 in the unilateral group, 8 in the bilateral group). Two patients died perioperatively (2.4%). Overall, LVRS improved forced expiratory volume in 1 s by 8.3% after 3 and 12.5% after 6 months postoperatively compared to baseline. Bilateral surgery presented significantly superior forced expiratory volume in 1 s improvement than unilateral approach at both 3 (29.2% versus 2.9%; *P* = 0.0010) and 6 months (21.5% versus 3%; *P* = 0.0310) postoperatively. Additionally, it reduced hyperinflation (residual volume) by 23.1% after 3 months and by 17.5% after 6 months, compared to reductions of 16% and 9.1% in the unilateral group.

**CONCLUSIONS:**

Bilateral approach resulted in better functional outcomes 3 and 6 months postoperatively compared to unilateral surgery.

## Graphical abstract

**Figure:**
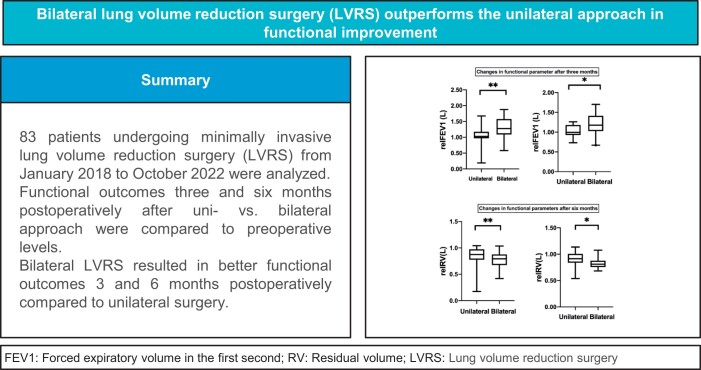

## INTRODUCTION

Chronic obstructive pulmonary disease (COPD) has a high prevalence and is one of the most common causes of death worldwide. Severe COPD is associated with pulmonary emphysema characterized by rarefication of the pulmonary parenchymal structure and hyperinflation [1]. Whereas optimal medical treatment and pulmonary rehabilitation can temporarily alleviate symptoms in these highly respiratory-impaired patients, a notable number of patients with advanced emphysema show disease progression [2]. In patients with end-stage disease, a lung transplant is an established surgical treatment option but not every patient with emphysema is eligible for a transplant. Bronchoscopic and surgical lung volume reduction (LVR) are also established treatments for patients with end stage emphysema either as a palliative measure or to delay the need for a lung transplant [2, 3].

The goal of LVR is to reduce hyperinflation in patients with emphysema by improving diaphragmatic function and respiratory mechanics [1]. Lung volume reduction surgery (LVRS), first used in the 1950s, showed functional improvement in patients with pulmonary emphysema but had a high mortality rate [4]. The National Emphysema Treatment Trial (NETT) was a prospective multicentre randomized controlled trial that compared optimal medical treatment with additional LVRS and defined risk factors to identify patients who would benefit from LVRS. The results showed that LVRS did not increase the mortality rate in selected patients [4]. Subsequent studies confirmed these findings and concluded better functional outcomes and overall survival after bilateral intervention compared to a unilateral approach [5, 6]. However, in these early studies, lung volume reduction was performed via open surgery. Since then, the LVR technique via surgical approaches has been substantially developed [1]. In selected patients with no interlobar collateral ventilation and with heterogeneous emphysema, bronchoscopic LVR with endobronchial valve placement is an alternative treatment to LVRS. In a recently published prospective randomized trial, functional outcome after LVRS was not superior to bronchoscopic LVR in patients suitable for both treatment approaches. However, in this study, LVRS was performed exclusively unilaterally [7]. Data regarding functional comparisons after unilateral or bilateral LVRS in the era of different alternative LVR procedures are scarce. Our goal was to investigate whether bilateral LVRS results in superior functional outcomes and potentially more postoperative complications with longer inpatient and intensive care unit stays compared to a unilateral approach in carefully selected patients who were evaluated for surgical and bronchoscopic LVR and were discussed in a multidisciplinary emphysema team meeting.

## MATERIALS AND METHODS

### Ethical statement

The study was approved by the institutional ethics committee of the University Hospital Essen (21–9856-BO). Study-specific informed patient consent was waived due to the retrospective study design. Of note, all patients provided written informed consent for utilization of clinical data for scientific studies and to be included in the European Society of Thoracic Surgeons database.

### Preoperative assessment and patient selection

This retrospective single-centre study included all consecutive emphysema patients who underwent LVRS from January 2018 to October 2022. Patients with a unilateral approach and a previous contralateral bronchoscopic or surgical intervention were excluded (*n* = 17). All patients were discussed in a weekly multidisciplinary emphysema team meeting.

Patients underwent standardized preoperative functional assessment. Patients with forced expiratory volume in 1 s (FEV1) >20% and <40%, diffusing capacity of the lung for carbon monoxide (DLCO) >20% and <40%, residual volume (RV) >160%, RV/total lung capacity (TLC) >0.58, TLC >100% and a walking distance of 150–450 metres in the 6-minute walk test (MWT) qualified for LVR. Each patient received high-resolution computed tomography to assess emphysema visually and to exclude other pulmonary pathologies like fibrosis. Transthoracic echocardiography was performed to exclude cardiac pathologies and pulmonary hypertension. If pulmonary hypertension was suspected, echocardiography was followed by right-heart catheterization. Quantitative computed tomography with Chartis measurements and lung perfusion scans (Chartis System, Pulmonx, Inc., Redwood City, CA, USA) were performed to decide between LVRS and bronchoscopic LVR. Quantitative computed tomography provides information about the completeness of the fissure in percent, lobar volume and a quantification of the emphysematous changes of each lobe. Lung perfusion scans help to determine the treatment target area. Collateral ventilation was assessed by bronchoscopic Chartis measurements and by a proprietary quantitative analysis of high resolution CT imaging data (StratX Lung Assessment Report, Pulmonx, Redwood City, CA, USA). The interdisciplinary emphysema board made treatment decisions by applying the same criteria throughout the observation period.

### Surgical technique

The routine approach for LVRS was via video-assisted thoracoscopic surgery (VATS). After dividing the pulmonary ligament and dissecting adhesions if present, apical crescent-shaped wedge resection was performed by applying a buttressed linear stapler. Patients routinely received two 24 Fr chest tubes, and no suction was applied on the chest tubes. In 2019, the institution changed from a unilateral to a bilateral LVRS as the method of choice due to a perceived advantage of the bilateral approach. Consequently, the choice of performing unilateral or bilateral surgery depended on the date of the operation.

### Follow-Up

Follow-up investigations, including a pulmonary function test and chest radiography, were performed 3 and 6 months postoperatively.

### Statistics

For continuous variables, a Student *t*-test was performed to compare groups if they were normally distributed. Parameters with non-Gaussian distribution were compared using a non-parametric Mann-Whitney test. Categorical data were compared using the Fisher exact or the χ^2^ test. P-values <0.05 were considered significant. All statistical analyses were conducted with GraphPad Prism 8.0 software (GraphPad Software, Boston, MA, USA).

## RESULTS

**Figure 1:**
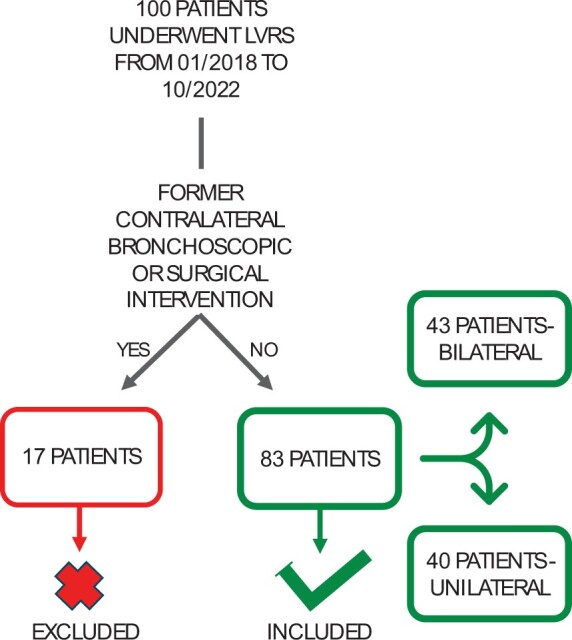
Patient recruitment. From 100 patients who underwent lung volume reduction surgery between January 2018 and October 2022, a total of 17 patients had previous contralateral interventions and were excluded. A total of 83 patients were included in the study with 43 patients in the bilateral and 40 patients in unilateral group. LVRS: lung volume reduction surgery.

Between January 2018 and October 2022, a total of 100 patients underwent LVRS at our institution. Seventeen patients were excluded from the study due to previous interventions. Finally, 83 patients were included in our study (Table 1). Bilateral LVRS was performed in 43 patients. Forty patients received unilateral LVRS (Fig. 1). Of these, 43 were male and 40 were female. The mean age of the cohort was 62.5 ± 7.9 years.

**Table 1:** Baseline characteristics and preoperative functional parameters grouped by operative approach

		Total		Unilateral		Bilateral		*P*-value
		*n*	Mean ± SD (95% CI)	*n*	Mean ± SD (95% CI)	*n*	Mean ± SD (95% CI)	
Gender	Male	43	–	21	–	22	–	>0.99
	Female	40	–	19	–	21	–	
Age		83	62.5 ± 7.9	40	61.6 ± 7.9	43	63.3 ± 7.9	0.35
			(60.8–64.2)		(59.1–64.1)		(60.9–65.7)	
FEV1 (%)		83	28.3 ± 5.3	40	28.5 ± 5.9	43	28.1 ± 4.7	0.93
			(27.1–29.4)		(26.2–30.4)		(26.6–29.5)	
FEV1 (L)		83	0.8 ± 0.2	40	0.9 ± 0.3	43	0.8 ± 0.2	0.90
			(0.8–0.9)		(0.8–1.0)		(0.8–0.9)	
DLCO (%)		81	28.3 ± 7.2	38	27.3 ± 7.5	43	29.1 ± 6.9	0.21
			(26.7–29.9)		(24.8–29.8)		(27.0–31.2)	
TLC (L)		83	8.7 ± 1.6	40	8.6 ± 1.6	43	8.7 ± 17	0.70
			(8.3–9.0)		(8.1–9.1)		(8.2–9.3)	
TLC (%)		83	142.0 ± 23.4	40	143.3 ± 30.5	43	140.7 ± 14.0	0.72
			(136.9–147.1)		(133.5–153.0)		(136.4–145.0)	
RV (L)		83	5.9 ± 1.4	40	5.9 ± 1.5	43	6.0 ± 1.3	0.96
			(5.6–6.2)		(5.5–6.4)		(5.6–6.4)	
RV (%)		83	267.3 ± 61.2	40	271.9 ± 78.3	43	262.9 ± 39.7	0.83
			(253.9–280.6)		(246.9–297.0)		(250.7–275.1)	
6-MWT (m)		78	303.9 ± 84.7	35	322.4 ± 86.9	43	288.8 ± 79.5	0.086
			(285.0–322.9)		(292.6–352.3)		(264.4–313.3)	

6-MWT: 6-minute walk test; CI: confidence interval; DLCO: diffusion capacity of the lungs for carbon monoxide; FEV1: forced expiratory volume in 1 s; L: liter; RV: residual volume; SD: standard deviation; TLC: total lung capacity.

Baseline demographic and functional parameters were comparable between the 2 groups (Table 2). The mean FEV1 was 28.3% ± 5.3 predicted; the DLCO was 28.3% ± 7.2 predicted; TLC was 142.0% ± 23.4 predicted; and RV was 267.3% ± 61.2 predicted. In 78 patients, the 6-minute walk test (6-MWT) results were available with a mean of 303.9 ± 84.7 metres. The preoperative functional parameters were grouped by the side of the operation, and no significant differences were found in FEV1 (L) (*P* = 0.9008), FEV1 (%) (*P* = 0.9330), DLCO (%) (*P* = 0.2051), TLC (L) (*P* = 0.7016), TLC (%) (*P* = 0.7218), RV (L) (*P* = 0.9620), RV (%) (*P* = 0.38260) and in the 6-MWT (*P* = 0.0861) between the uni- and bilateral approaches.

**Table 2:** Changes in functional parameters grouped by operative approach after 3 months

relFEV1 (L)	*n*	Mean ± SD	95% CI	*P-*value
All	58	1.183 ± 0.3717	1.085–1.281	–
Unilateral	24	1.029 ± 0.2997	0.9025–1.156	**0.0010**
Bilateral	34	1.292 ± 0.3828	1.158–1.426	

relRV (L)	*n*	Mean ± SD	95% CI	*P-*value

All	58	0.7988 ± 0.1974	0.7469–0.8506	–
Unilateral	24	0.8403 ± 0.2060	0.7533–0.9273	**0.0096**
Bilateral	34	0.7694 ± 0.1886	0.7036–0.8352	

CI: confidence interval; FEV1: forced expiratory volume in 1 s; rel: relative; RV: residual volume; SD: standard deviation.

In 79 patients (95%), the surgical procedure was performed by VATS. Four (5%) patients required conversion to thoracotomy due to severe adhesions or technical issues. The most common postoperative complication was prolonged air leak beyond 5 days (*n* = 19; 22.9%). Among these patients, 10 underwent a reoperation for fistula closure with 5 patients from the unilateral group and 5 from the bilateral group (*P* > 0.999). Perioperative death occurred in 2 patients (2.4%). The first patient presented after bilateral LVRS with a prolonged air leak and postoperative delirium. The patient died of sudden cardiac death on postoperative day 5. The second patient received bilateral LVRS and died 1 month postoperatively of pneumonia and severe sepsis. The mean hospital stay was 11.8 ± 7.0 days. Patients who had bilateral LVRS had significantly shorter inpatient stays compared to patients with unilateral LVRS (11.0 ± 7.0 vs 12.6 ± 6.9; *P* = 0.0497). Duration of drainage (*P* = 0.1052) and intensive care unit stay (*P* = 0.0836) did not differ significantly between the 2 groups (Supplementary Material, Table S7). In the bilateral group, the date of the last removed drain was considered.

**Figure 2:**
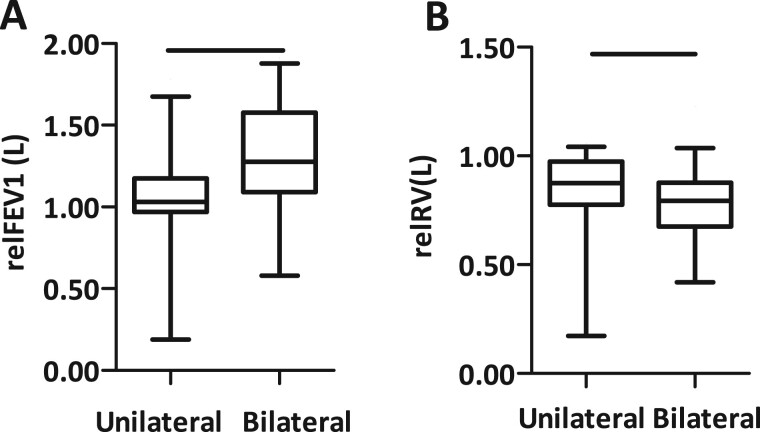
Functional outcome after 3 months. (**A**) After bilateral lung volume reduction surgery, forced expiratory volume in 1 s improved by 29.2% whereas unilateral surgery improved forced expiratory volume in 1 s by 2.9% (*P* = 0.0010). (**B**) Unilateral lung volume reduction surgery resulted in a reduction of 16.0% and bilateral lung volume reduction surgery of 23.1% of hyperinflation after 3 months (*P* = 0.0096). relFEV1: relative forced expiratory volume in 1 s; relRV: relative residual volume.

Changes in functional parameters after unilateral or bilateral LVRS were analysed 3 months postoperatively. LVRS improved FEV1 by 18.3% 3 months postoperatively and reduced RV by 20.2%. Bilateral operations led to significantly better functional outcomes than unilateral operations. For example, FEV1 improved by 2.9% after unilateral and 29.2% after bilateral operations (*P* = 0.0010). Bilateral LVRS led to a decrease of 23.1% in RV, whereas unilateral operations reduced RV by 16.0% (*P* = 0.096) (Fig. 2, Table 2).

**Figure 3:**
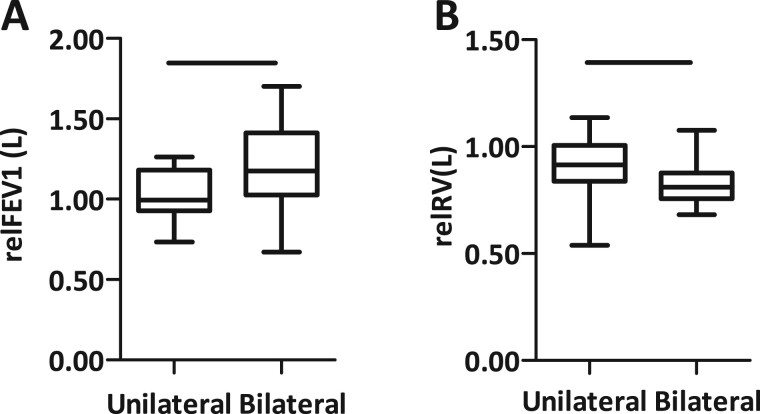
Functional outcome after 6 months. (**A**) Bilateral lung volume reduction surgery improved forced expiratory volume in 1 s by 21.5% and unilateral surgery by 3.0% (*P* = 0.0310). (**B**) Hyperinflation decreased after unilateral surgery by 9.0% and after bilateral lung volume reduction surgery by 17.5% (*P* = 0.0103). relFEV1: relative forced expiratory volume in 1 s; rel RV: relative residual volume.

Six months postoperatively, the overall cohort presented an improvement of 12.5% in FEV1 and a reduction of 13.5% in RV after LVRS. Again, better outcomes were observed after bilateral LVRS than after unilateral operations. Unilateral operations resulted in an FEV1 increase of 3.0% and an RV decrease of 9.0% compared to preoperative levels. After bilateral LVRS, the improvement in FEV1 was 21.5%, and RV decreased 17.6%. The difference between a unilateral and a bilateral operation was significant for FEV1 (*P* = 0.0310) as well as for RV (*P* = 0.0103) (Fig. 3, Table 3).

**Table 3.** Changes in functional parameters grouped by operative approach after 6 months

relFEV1 (L)	*n*	Mean ± SD	95% CI	*P-*value
All	37	1.125 ± 0.2389	1.045–1.204	/
Unilateral	18	1.030 ± 0.1568	0.9518–1.108	**0.0310**
Bilateral	19	1.215 ± 0.2710	1.084–1.345	

relRV (L)	*n*	Mean ± SD	95% CI	*P-*value

All	38	0.8652 ± 0.1314	0.8220–0.9084	/
Unilateral	18	0.9100 ± 0.1460	0.8374–0.9826	**0.0103**
Bilateral	20	0.8249 ± 0.1046	0.7759–0.8739	

CI: confidence interval; FEV1: forced expiratory volume in 1 s; L: liter; rel: relative; RV: residual volume; SD: standard deviation.

We considered age, gender and 6-MWT as possible confounders. No differences were observed, except for gender, where the FEV1 difference was significant after 6 months. Nonetheless, the number of patients was too small for further subgroup analyses. The other parameters examined did not significantly influence the outcome (Supplementary Material, Tables S1–S6).

## DISCUSSION

LVR surgery is an established palliative treatment for patients with severe pulmonary emphysema and requires careful patient selection [4, 8–11]. Several studies investigated the effects of LVRS in patients with advanced COPD and demonstrated significant functional improvement in carefully selected patients and underlined the importance of accurate patient selection as well as clinical experience for optimal pre- and postoperative management in this highly vulnerable patient cohort [1, 4, 8–11].

In our study, 79 patients received LVRS by VATS. Bilateral LVRS was only performed via VATS. The first results of studies investigating bilateral LVRS were published in the 1990s. Cooper *et al.* described the results of 150 patients after bilateral LVRS through a median sternotomy. The authors described the median sternotomy as an ultimate muscle-sparing approach with excellent bilateral exposure [12]. Ciccone *et al.* included 250 patients in their study who also underwent bilateral LVRS through a median sternotomy [8]. An additional study evaluated the efficiency of a thoracoscopic but unilateral approach to LVRS and found improvement in pulmonary mechanics and functional parameters [13]. Although thoracoscopic LVRS is used more frequently now, one-stage thoracoscopic bilateral LVRS is still not standard in most centres.

The main postoperative complication in our cohort was prolonged air leak, which occurred in 19 patients. In 10 patients, reoperation was required for fistula closure. In the National Emphysema Treatment Trial (NETT) and further prior studies, prolonged air leak was the most reported complication in up to 45% of patients after LVRS [8, 12, 14, 15]. Thus, our data are in line with those of earlier studies. Notably, in our study, bilateral thoracoscopic LVRS was not associated with higher rates of prolonged air leak.

In our overall cohort, the mean hospital stay was 11.8 ± 7.0 days. Interestingly, patients who received bilateral LVRS had significantly shorter inpatient stays compared to patients who received unilateral LVRS. Consequently, our study indicates that bilateral thoracoscopic LVRS is not associated with longer hospital stays. On the contrary, patients recovered more quickly after bilateral LVRS than after unilateral LVRS. Cooper *et al.*, who performed bilateral LVRS through a median sternotomy, described similar mean hospital stays with 13.5 days in the first 100 patients and with 10 days in the last 50 patients [12]. Sadler *et al.* also reported a mean hospital stay of 14.3 days [15]. In this context, chest tube duration rather than the surgical approach determines the most likely hospital stay. Thus, no notable difference was observed between prior studies and our studies regarding inpatient stay after LVRS.

Some studies compared functional outcomes with unilateral and bilateral LVRS. They differed as to surgical approach and as to a staged or one-staged method. Common to all these studies was a benefit in overall survival [11] and better functional results after bilateral surgery [5, 6, 12]. Our study results were in line with those of former studies. We showed that unilateral and bilateral LVRS improved functional parameters 3 and 6 months after the operation but with better functional results after bilateral LVRS. The majority of prior studies were performed at a time when interventional LVR methods were not present and established like they are today. Consequently, patients were not evaluated carefully regarding surgical or bronchoscopic LVR. Meanwhile, the state-of-the-art endoscopic LVR procedures require interdisciplinary experience to assess whether a patient is an appropriate candidate for a surgical or a bronchoscopic LVR [1]. Recently published data focused on patients suitable for both treatment approaches and found surgical LVR not to be superior to bronchoscopic LVR. Of note, patients in this study underwent exclusively unilateral LVRS [7]. Our data show that, in the era of multidisciplinary emphysema-board decision making, bilateral LVRS is superior to unilateral LVRS. Based on our results, prospective studies comparing bilateral LVRS with interventional LVR in patients suitable for surgical and bronchoscopic LVR are of significant interest.

### Limitations

The limitations of our study are the single-centre design and the non-randomized setting with a historical control group. However, uniform decision making in the single-centre setting and comparable baseline values ensure the validity of the results, which, however, have to be confirmed by prospective randomized data. Unfortunately, some of the patients were lost to follow-up after 3 and 6 months, but the proportion of lost patients was similar in both groups. The fact that a significant number of patients went to their local pulmonologists for further care contributed to the high number of patients lost to follow-up. Other limitations include the limited number of patients, which can be attributed to the fact that patients with LVRS are evaluated carefully and the procedure is performed less frequently overall. Nonetheless, to the best of our knowledge, our study is the first to demonstrate that one-staged bilateral LVRS leads to better functional outcome compared to the unilateral approach.

## CONCLUSION

Our study shows reproducible functional improvement after LVRS in carefully selected patients with end stage emphysema. Bilateral thoracoscopic LVR resulted in significantly better functional outcomes compared to unilateral surgery.

## Supplementary Material

ivae169_Supplementary_Data

## Data Availability

The data underlying this article will be shared on reasonable request to the corresponding author.

## ABBREVIATIONS

6-MWT: 6-min walk test
COPD: Chronic obstructive pulmonary disease
DLCO: Diffusing capacity of the lung for carbon monoxide
FEV1: Forced expiratory volume in 1 s
LVR: Lung volume reduction
LVRS: Lung volume reduction surgery
RV: Residual volume
TLC: Total lung capacity
VATS: Video-assisted thoracoscopic surgery
