# Supplementary material for: Bilateral lung volume reduction surgery outperforms the unilateral approach in functional improvement
Source: Interdiscip Cardiovasc Thorac Surg. 2024 Oct 1;39(4):ivae169. doi: 10.1093/icvts/ivae169 (PMC11502496; doi:10.1093/icvts/ivae169)
Supplement: ivae169_Supplementary_Data [file ivae169_supplementary_data.docx]

**SUPPLEMENTARY DATA**

**Table S1**. Changes in functional parameters grouped by gender after three months.

| **relFEV1 (L)** | **n** | **MEAN ±SD** | **95% CI** | **p** |
| --- | --- | --- | --- | --- |
| ALL | 58 | 1.2 ± 0.37 | 1.1 – 1.3 | / |
| Male | 30 | 1.2 ± 0.41 | 1.0 – 1.3 | 0.4842 |
| Female | 28 | 1.2 ± 0.33 | 1.1 – 1.3 |  |
| **relRV (L)** | **n** | **MEAN ±SD** | **95% CI** | **p** |
| ALL | 58 | 0.8 ± 0.2 | 0.75 – 0.85 | / |
| Male | 30 | 0.8 ± 0.2 | 0.72 – 0.87 | 0.9846 |
| Female | 28 | 0.8 ± 0.2 | 0.73 – 0.88 |  |

**Table S2.** Changes in functional parameters grouped by gender after six months.

| **relFEV1 (L)** | **n** | **MEAN ±SD** | **95% CI** | **p** |
| --- | --- | --- | --- | --- |
| ALL | 37 | 1.1 ± 0.24 | 1.0 – 1.2 | / |
| Male | 18 | 1.1 ± 0.28 | 0.92 – 1.2 | **0.0456** |
| Female | 19 | 1.2 ± 0.18 | 1.1 – 1.3 |  |
| **relRV (L)** | **n** | **MEAN ±SD** | **95% CI** | **p** |
| ALL | 38 | 0.87 ± 0.13 | 0.82 – 0.91 | / |
| Male | 18 | 0.89 ± 0.13 | 0.83 – 0.96 | 0.2896 |
| Female | 20 | 0.84 ± 0.13 | 0.73 – 0.90 |  |

**Table S3.** Changes in functional parameters grouped by age after three months.

| **relFEV1 (L)** | **n** | **MEAN ±SD** | **95% CI** | **p** |
| --- | --- | --- | --- | --- |
| ALL | 58 | 1.2 ± 0.37 | 1.1 – 1.3 | / |
| </=63 | 30 | 1.1 ± 0.32 | 1.0 – 1.3 | 0.3353 |
| >63 | 28 | 1.2 ± 0.42 | 1.1 – 1.4 |  |
| **relRV (L)** | **n** | **MEAN ±SD** | **95% CI** | **p** |
| ALL | 58 | 0.8 ± 0.2 | 0.75 – 0.85 | / |
| </=63 | 30 | 0.8 ± 0.18 | 0.73 – 0.87 | 0.8319 |
| >63 | 28 | 0.8 ± 0.2 | 0.71 – 0.88 |  |

**Table S4.** Changes in functional parameters grouped by age after six months.

| **relFEV1 (L)** | **n** | **MEAN ±SD** | **95% CI** | **p** |
| --- | --- | --- | --- | --- |
| ALL | 37 | 1.1 ± 0.24 | 1.0 – 1.2 | / |
| </=63 | 26 | 1.1 ± 0.23 | 1.0 – 1.2 | 0.3490 |
| >63 | 11 | 1.2 ± 0.26 | 1.0 – 1.4 |  |
| **relRV (L)** | **n** | **MEAN ±SD** | **95% CI** | **p** |
| ALL | 38 | 0.87 ± 0.13 | 0.82 – 0.91 | / |
| </=63 | 27 | 0.88 ± 0.12 | 0.83 – 0.93 | 0.3560 |
| >63 | 11 | 0.82 ± 0.15 | 0.72 – 0.93 |  |

**Table S5.** Changes in functional parameters grouped by 6-MWT after three months.

| **relFEV1 (L)** | **n** | **MEAN ±SD** | **95% CI** | **p** |
| --- | --- | --- | --- | --- |
| ALL | 55 | 1.2 ± 0.38 | 1.1 – 1.3 | / |
| </=305m | 26 | 1.2 ± 0.31 | 1.1 – 1.3 | 0.7602 |
| >305m | 29 | 1.2 ± 0.44 | 0.99 – 1.3 |  |
| **relRV (L)** | **n** | **MEAN ±SD** | **95% CI** | **p** |
| ALL | 55 | 0.80 ± 0.2 | 0.74 – 0.85 | / |
| </=305m | 26 | 0.83 ± 0.14 | 0.78 – 0.89 | 0.5441 |
| >305m | 29 | 0.77 ± 0.24 | 0.68 – 0.85 |  |

**Table S6.** Changes in functional parameters grouped by 6-MWT after six months.

| **relFEV1 (L)** | **n** | **MEAN ±SD** | **95% CI** | **p** |
| --- | --- | --- | --- | --- |
| ALL | 35 | 1.1 ± 0.24 | 1.0 – 1.2 | / |
| </=305m | 16 | 1.1 ± 0.25 | 0.94 – 1.2 | 0.0763 |
| >305m | 19 | 1.2 ± 0.23 | 1.1 – 1.3 |  |
| **relRV (L)** | **n** | **MEAN ±SD** | **95% CI** | **p** |
| ALL | 36 | 0.86 ± 0.13 | 0.82 – 0.9 | / |
| </=305m | 16 | 0.88 ± 0.12 | 0.81 – 0.94 | 0.5187 |
| >305m | 20 | 0.85 ± 0.13 | 0.79 – 0.91 |  |

**Table S7.** Postoperative Outcome

|  | **ALL** | | **UNILATERAL** | | **BILATERAL** | | **p** |
| --- | --- | --- | --- | --- | --- | --- | --- |
|  | **n** | **Mean** ± SD (95% CI) | **n** | **Mean** ± SD (95% CI) | **n** | **Mean** ± SD (95% CI) |  |
| **ICU-STAY** | 58 | 1.7 ± 3.9  (0.65 – 2.7) | 18 | 0.89 ± 0.47  (0.65 – 1.1) | 40 | 2.1 ± 4.7  (0.54 – 3.6) | 0.0836 |
| **LENGTH OF DRAINAGE** | 81 | 7.5 ± 6.3  (6.1 – 8.9) | 40 | 8.2 ±6.6  (6.1 – 10) | 41 | 6.8 ± 6.1  (4.9 – 8.7) | 0.1052 |
| **HOSPITAL STAY** | 83 | 11.8 ± 7.0 | 40 | 12.6 ± 7.0 | 43 | 11.1 ± 7.0 | **0.0497** |

**Table S8.** Baseline characteristics and preoperative functional parameters grouped by operative approach.

|  | | **Total** | | **Unilateral** | | **Bilateral** | | **p** |
| --- | --- | --- | --- | --- | --- | --- | --- | --- |
|  |  | **n** | **Median (IQR)** | **n** | **Median (IQR)** | **n** | **Median (IQR)** |  |
| Gender | Male | 43 | - | 21 | - | 22 | - | >0.9999 |
|  | Female | 40 | - | 19 | - | 21 | - |  |
| Age | | 83 | 63 (11) | 40 | 62 (11) | 43 | 64 (11) | 0.3524 |
| FEV1 (%) | | 83 | 28 (6) | 40 | 28 (6) | 43 | 28 (6) | 0.9330 |
| FEV1 (L) | | 83 | 0.82 (0.28) | 40 | 0.87 (0.2) | 43 | 0.81 (0.29) | 0.9008 |
| DLCO (%) | | 81 | 27 (10) | 38 | 27 (10) | 43 | 28 (11) | 0.2051 |
| TLC (L) | | 83 | 8.6 (2.4) | 40 | 8.5 (2.1) | 43 | 8.6 (2.5) | 0.7016 |
| TLC (%) | | 83 | 138 (24) | 40 | 138 (28) | 43 | 138 (25) | 0.7218 |
| RV (L) | | 83 | 5.7 (1.4) | 40 | 5.7 (1.2) | 43 | 5.7 (1.7) | 0.9620 |
| RV (%) | | 83 | 256 (53) | 40 | 256 (66) | 43 | 255 (40) | 0.8260 |
| 6-MWT (m) | | 78 | 305 (131) | 35 | 320 (115) | 43 | 290 (135) | 0.0861 |

**Table S9.** Changes in functional parameters grouped by operative approach after three months.

| **relFEV1 (L)** | **n** | **Median (IQR)** | **p** |
| --- | --- | --- | --- |
| ALL | 58 | 1.144 (0.415) | / |
| Unilateral | 24 | 1.031 (0.2043) | **0.0010** |
| Bilateral | 34 | 1.276 (0.487) |  |
| **relRV (L)** | **n** | **Median (IQR)** | **p** |
| ALL | 58 | 0.8280 (0.1869) | / |
| Unilateral | 24 | 0.8741 (0.1978) | **0.0096** |
| Bilateral | 34 | 0.7936 (0.2018) |  |

**Table S10.** Changes in functional parameters grouped by operative approach after six months.

| **relFEV1 (L)** | **n** | **Median (IQR)** | **p** |
| --- | --- | --- | --- |
| ALL | 37 | 1.133 (0.3172) | / |
| Unilateral | 18 | 0.944 (0.2533) | **0.0310** |
| Bilateral | 19 | 1.176 (0.386) |  |
| **relRV (L)** | **n** | **Median (IQR)** | **p** |
| ALL | 38 | 0.8477 (0.1457) | / |
| Unilateral | 18 | 0.9147 (0.1682) | **0.0103** |
| Bilateral | 20 | 0.8104 (0.1211) |  |
